# Knowledge, attitudes, and practice patterns related to vocal hygiene among conservatory students in China

**DOI:** 10.3389/fpubh.2026.1942292

**Published:** 2026-10-07

**Authors:** Miao Sun, Lin Zhou

**Affiliations:** 1 School of Music, Nanjing Normal University, Nanjing, Jiangsu, China; 2 Department of Popular Function, Sichuan Conservatory of Music, Chengdu, Sichuan, China

**Keywords:** attitudes, conservatory students, knowledge, practices, vocal hygiene, voice symptoms

## Abstract

**Objective:**

To assess vocal hygiene knowledge, attitudes, voice-related practices, and educational needs among conservatory students in mainland China. Self-reported voice symptoms, previous clinician-diagnosed voice disorders, and symptom-management behaviours were evaluated as secondary outcomes.

**Methods:**

This cross-sectional study used a self-administered online questionnaire and convenience sampling. A total of 315 conservatory students were included. The questionnaire comprised six sections assessing participant characteristics, voice disorder history and symptoms, vocal hygiene knowledge, attitudes, voice-related practices, and educational needs. Correlation analyses, multivariable linear regression, and separate multivariable logistic regression models for nine voice-related practices were performed.

**Results:**

The median knowledge score was 8 (interquartile range [IQR], 7–9), and the median attitude score was 7 (IQR, 5–8). Frequent whispering had the lowest correct-response rate (26.0%) and the highest proportion of “Do not know” responses (40.0%). Knowledge and attitude scores were weakly positively correlated (Spearman’s *ρ* = 0.143, *p* = 0.011). Frequent throat clearing was reported by 44.1% of participants, whereas 41.3% reported warming up before singing and 27.9% reported cooling down afterwards. Each one-point increase in knowledge score was associated with higher odds of warming up before singing (adjusted odds ratio [aOR] = 1.28, 95% confidence interval [CI], 1.11–1.47; false discovery rate-adjusted *p* = 0.006), but knowledge score was not associated with any of the seven potentially harmful practices. Each additional recent voice symptom was associated with higher odds of all seven potentially harmful practices (aOR range, 1.25–1.97; all false discovery rate-adjusted *p* ≤ 0.016). As secondary findings, 196 participants (62.2%) reported at least one recent voice symptom, but only 21 of these participants (10.7%) sought care from a school medical institution or hospital. Overall, 273 participants (86.7%) expressed interest in obtaining further vocal hygiene information.

**Conclusion:**

Conservatory students demonstrated generally favourable vocal hygiene knowledge and attitudes, but specific knowledge gaps and limited translation of knowledge into appropriate voice-related practices remained. These findings identify priorities for targeted, practice-oriented vocal health content and clearer healthcare-seeking pathways in conservatory settings.

## Introduction

Voice is essential to many occupations, particularly those in which professional performance depends on sustained, precise, and reliable voice production (1). Potentially harmful voice-use behaviours, including excessive or prolonged voice use, shouting, habitual throat clearing, and speaking or singing with inappropriate loudness or effort, have been associated with voice complaints such as hoarseness, vocal fatigue, reduced vocal range, insufficient vocal intensity, and diminished vocal efficiency (2–4). Singers are particularly vulnerable because their professional and artistic performance depends on fine control of pitch, intensity, timbre, and endurance. A systematic review and meta-analysis reported a pooled prevalence of self-reported dysphonia of 46.09% among singers, although prevalence varied substantially across populations and was lower among singing students than among established singers and singing teachers (5). Voice problems may consequently interfere with training, performance, occupational participation, and psychosocial well-being (6).

Conservatory students represent a distinct subgroup of professional voice users in training. Their vocal load may include individual practice, one-to-one lessons, ensemble rehearsals, performances, examinations, and substantial everyday speaking, often while vocal technique and physiological coordination are still developing. They may also have less control over rehearsal schedules, repertoire, recovery periods, and performance-related voice use than established professionals. Studies of singers in training have demonstrated considerable variation in their knowledge of vocal anatomy, pathology, and appropriate care-seeking, while even relatively short periods of intensive singing may increase self-perceived vocal fatigue and complaints (7, 8). These characteristics indicate that evidence obtained from teachers, broadcasters, lawyers, or established professional singers cannot necessarily be generalised to conservatory students.

Vocal hygiene refers to behavioural, environmental, and health-related measures intended to maintain vocal function and reduce avoidable phonotraumatic exposure. These measures commonly include adequate hydration, management of vocal load and recovery, reduction of habitual shouting and throat clearing, avoidance of smoke and other laryngeal irritants, appropriate warm-up and cool-down routines, and timely assessment of persistent voice symptoms (2, 9). Vocal hygiene is generally delivered as a preventive strategy and as an indirect, foundational component of voice care. Evidence accumulated over the past two decades supports its clinical relevance, although outcomes vary according to the population, intervention content, and outcome measure. A recent systematic review of professional voice users found that vocal hygiene education was associated with positive outcomes in several studies but was generally more effective when combined with direct voice therapy or voice training than when delivered alone (10). A systematic review focusing specifically on singers similarly reported improvements in measures such as maximum phonation time, vocal intensity, the Dysphonia Severity Index, and the use of vocal rest, while also emphasising the small number and limited sample sizes of available studies (11). Thus, vocal hygiene education is best understood as an evidence-informed baseline intervention that should be tailored to the individual’s vocal demands.

Studies conducted in other populations further suggest that voice-care knowledge does not necessarily translate into favourable attitudes or daily practices. Surveys have identified heterogeneous knowledge and treatment-related concerns among classical singers in training in the United States (7), insufficient knowledge of vocal health and hygiene among Brazilian student teachers (12), and substantial gaps in knowledge and attitudes among occupational voice users in India (13). However, such findings are shaped by professional demands, educational systems, access to voice-care services, and cultural understandings of voice. These factors are particularly relevant in China, where conservatory training may encompass Western classical singing, modern Chinese national or folk singing, and traditional Chinese operatic styles. Physiological and acoustic studies have identified differences between some Chinese and Western singing traditions in laryngeal position, resonance strategies, glottal behaviour, and register adjustment (14, 15). These differences demonstrate distinct vocal-production strategies, but current evidence does not establish that one tradition is inherently more damaging to the vocal folds than another. They instead underscore the need for culturally and genre-sensitive vocal hygiene education. Moreover, speech-language pathology exists but remains a developing discipline in China; consequently, the availability of specialised and multidisciplinary voice-care pathways may vary across settings (16).

Despite growing international attention to singers’ vocal health, published evidence concerning the knowledge, attitudes, practices, and perceived educational needs of conservatory students in mainland China remains limited. The present cross-sectional survey therefore aimed to assess conservatory students’ knowledge, attitudes, practices, and demands regarding vocal hygiene. The findings are intended to identify population-specific educational gaps and inform the development of culturally and pedagogically appropriate vocal hygiene programmes. The long-term goal is to support vocal health, performance sustainability, and vocal longevity among singers in training.

## Materials and methods

### Design and ethical approval

This cross-sectional study was conducted using a self-administered online questionnaire hosted on Wenjuanxing (https://www.wjx.cn/). Participants were recruited through convenience sampling from music conservatories in different regions of China. The study was approved by the Ethics Committee of Sichuan University (No. 2024 [385]). Electronic informed consent was obtained from all participants before they accessed the questionnaire.

### Questionnaire development

The initial questionnaire was developed with reference to previous studies and guidelines concerning vocal hygiene among singers and other professional voice users (7, 17–20). Singer-specific sources informed questions related to singing and vocal demands, whereas broader literature informed general vocal hygiene items. The draft was reviewed by three otolaryngologists from West China Hospital and an associate professor from Sichuan Conservatory of Music. Two otolaryngologists had more than 3 years of clinical experience, and the third held the professional title of associate chief physician.

The initial item pool comprised 35 individual items. One salient recommendation was to distinguish vocal hygiene knowledge from general singing-technique knowledge. Accordingly, five items concerning preferred breathing patterns, singing posture, abdominal state during quiet breathing, abdominal engagement during singing, and laryngeal position were removed because they did not directly assess vocal hygiene and could vary according to pedagogical approach. One hypothetical item concerning treatment preferences for vocal fold nodules or polyps was also removed during questionnaire consolidation because it did not directly assess the prespecified KAP domains. Following expert review, the revised questionnaire was pilot-tested among 20 eligible conservatory students to assess item clarity and comprehensibility. Pilot feedback resulted only in minor wording refinements. For example, the ambiguous response option “Observation” was revised to “Monitored the symptoms and waited for them to improve without treatment.” These refinements did not alter the meaning, scoring, or structure of the questionnaire. The responses of the 20 pilot participants were retained in the final analytic sample as a pragmatic measure to preserve the attainable sample size and study feasibility. The final questionnaire comprised 29 individual items presented as 16 numbered questions across six sections, with several questions containing multiple items or response options. The complete questionnaire is provided in Supplementary Appendix 1.

### Questionnaire content and scoring

Section I: participant characteristics. This section contained four questions concerning gender, age, singing style, and vocal range.

Section II: Voice disorders. This section collected information on self-reported voice disorders, previous treatment or professional consultation, voice symptoms and the actions they had taken in response, and self-rated voice quality. The number of recent voice symptoms was calculated by summing the seven predefined symptoms reported during the preceding month, yielding a symptom count ranging from 0 to 7. Clinical exposure was categorised as “No specific diagnosis reported,” “Diagnosed, not treated,” or “Diagnosed and treated.” The first category included participants who had never sought medical evaluation for a voice problem and those who reported that no voice disorder had been diagnosed. Participants reporting at least one clinician-diagnosed voice disorder were classified as treated or untreated according to whether they had received medication, surgery, or voice therapy. “No specific diagnosis reported” was the reference category. Voice quality was evaluated using a single-item self-rating scale ranging from 1 to 10.

Section III: Vocal hygiene knowledge. This section contained 10 items assessing knowledge of the effects of voice-related behaviours on vocal health. For each item, participants selected “Beneficial to vocal health,” “Harmful to vocal health,” or “Do not know.” A correct response was assigned 1 point, whereas an incorrect or “Do not know” response was assigned 0 points, yielding a total knowledge score ranging from 0 to 10.

Section IV: Attitudes toward vocal hygiene. This section contained five statements assessing attitudes toward vocal hygiene. Responses were recorded using a five-point Likert scale: strongly disagree, disagree, neutral, agree, and strongly agree. Responses were coded from −2 to 2, with higher values indicating a more favourable attitude: strongly disagree = −2, disagree = −1, neutral = 0, agree = 1, and strongly agree = 2. The five item scores were summed to obtain a total attitude score ranging from −10 to 10.

Section V: Voice-related practices. This section assessed participants’ actual voice-related practices using a multiple-response checklist containing nine behaviours: seven potentially harmful behaviours and two protective vocal hygiene behaviours. Participants selected all behaviours that applied to them.

Section VI: Vocal hygiene educational needs. Identifying unmet educational needs was a prespecified secondary objective of the survey and could inform subsequent vocal health education for conservatory students. This section assessed the content and delivery channels through which participants wished to receive further vocal hygiene education. Self-rated familiarity with vocal hygiene was also recorded as a separate awareness variable using five response categories: Never heard of it, Aware of the term but unfamiliar with its meaning, Somewhat familiar, Familiar, Very familiar.

### Participants and response-quality criteria

Eligible participants were students who attended music conservatories in Sichuan, Jiangsu, Heilongjiang, Hebei, Yunnan, Shanxi, Jiangxi, Shandong, or Gansu Province or in Beijing Municipality.

Questionnaires were excluded if any of the following criteria were met: (1) the participant was younger than 18 years; (2) either “I have never sought medical evaluation for a voice problem” or “I sought medical evaluation, but no voice disorder was diagnosed” was selected together with one or more specific voice disorder diagnoses; (3) “No voice symptoms” was selected together with one or more specific voice symptoms; or (4) “None of the above” was selected together with one or more listed voice-related practices. These combinations were prespecified as logically inconsistent. Of the 344 questionnaires received, 17 were excluded because the participants were younger than 18 years and 12 because of logically inconsistent responses, leaving 315 questionnaires for analysis.

### Statistical analysis

Continuous variables were assessed for normality using the Kolmogorov–Smirnov test. Normally distributed continuous variables are presented as means with standard deviations, and nonnormally distributed variables as medians with interquartile ranges. Categorical variables are presented as frequencies and percentages. Associations between categorical variables were examined using Pearson’s chi-square test or Fisher’s exact test. The distribution of symptom-management behaviours across symptom-count groups was assessed using an exact test. Spearman’s rank correlation was used to assess the associations between symptom count and self-rated voice quality and between the knowledge and attitude scores. Kendall rank correlation was used to assess the association between the knowledge score and the five-level self-rated familiarity variable. The multivariable linear regression model was applied to investigate factors associated with vocal hygiene knowledge. HC3 heteroscedasticity-robust standard errors were used, and regression coefficients (*β*) with 95% confidence intervals were reported. Each of the nine predefined voice-related practices was analysed as a binary outcome in a separate exploratory multivariable logistic regression model. Each model included the continuous knowledge score and clinical exposure and was adjusted for age, gender, singing style, and recent symptom count. Vocal range was not included in the multivariable models because the low-range category contained only six participants, all of whom were male, resulting in sparse data and substantial overlap with gender. Adjusted odds ratios (aORs) with 95% confidence intervals were reported. To account for multiple testing across the nine voice-related practice outcomes, *p* values were adjusted separately for each predictor using the Benjamini–Hochberg false discovery rate procedure. All analyses were performed using R version 4.5.0 (R Foundation for Statistical Computing, Vienna, Austria). All tests were two-sided. *p* < 0.05 was considered statistically significant; for analyses subject to multiplicity correction, statistical significance was assessed using the FDR-adjusted *p* value.

## Results

### Participant characteristics

The study included 315 participants, with a median age of 20 years (interquartile range [IQR], 19–21 years). Most participants were female (*n* = 192, 61.0%). Vocal-range distribution was associated with gender (Fisher’s exact test, *p* < 0.001), as was singing style (*χ*^2^ = 21.619, *p* < 0.001) (Table 1).

**Table 1:** Participant characteristics by gender.

Characteristic	Male (*n* = 123), *n* (%)	Female (*n* = 192), *n* (%)
Vocal range		
High	81 (65.9%)	167 (87.0%)
Middle	36 (29.3%)	25 (13.0%)
Low	6 (4.9%)	0 (0.0%)
Singing style		
Folk	22 (17.9%)	75 (39.1%)
Classical	90 (73.2%)	90 (46.9%)
Popular	11 (8.9%)	27 (14.1%)

Percentages were calculated within gender categories.

### History of voice disorders and voice symptoms

Among the participants, 176 had never sought medical evaluation for a voice problem, whereas 69 reported that no voice disorder had been diagnosed. Chronic laryngitis was the most frequently reported clinician-diagnosed voice disorder (*n* = 50, 15.9%); no cases of vocal fold paralysis or sulcus vocalis were reported (Figure 1A). Overall, 56 participants (17.8%) reported having previously received treatment for a voice disorder, including medication, surgery, or voice therapy.

**Figure 1:**
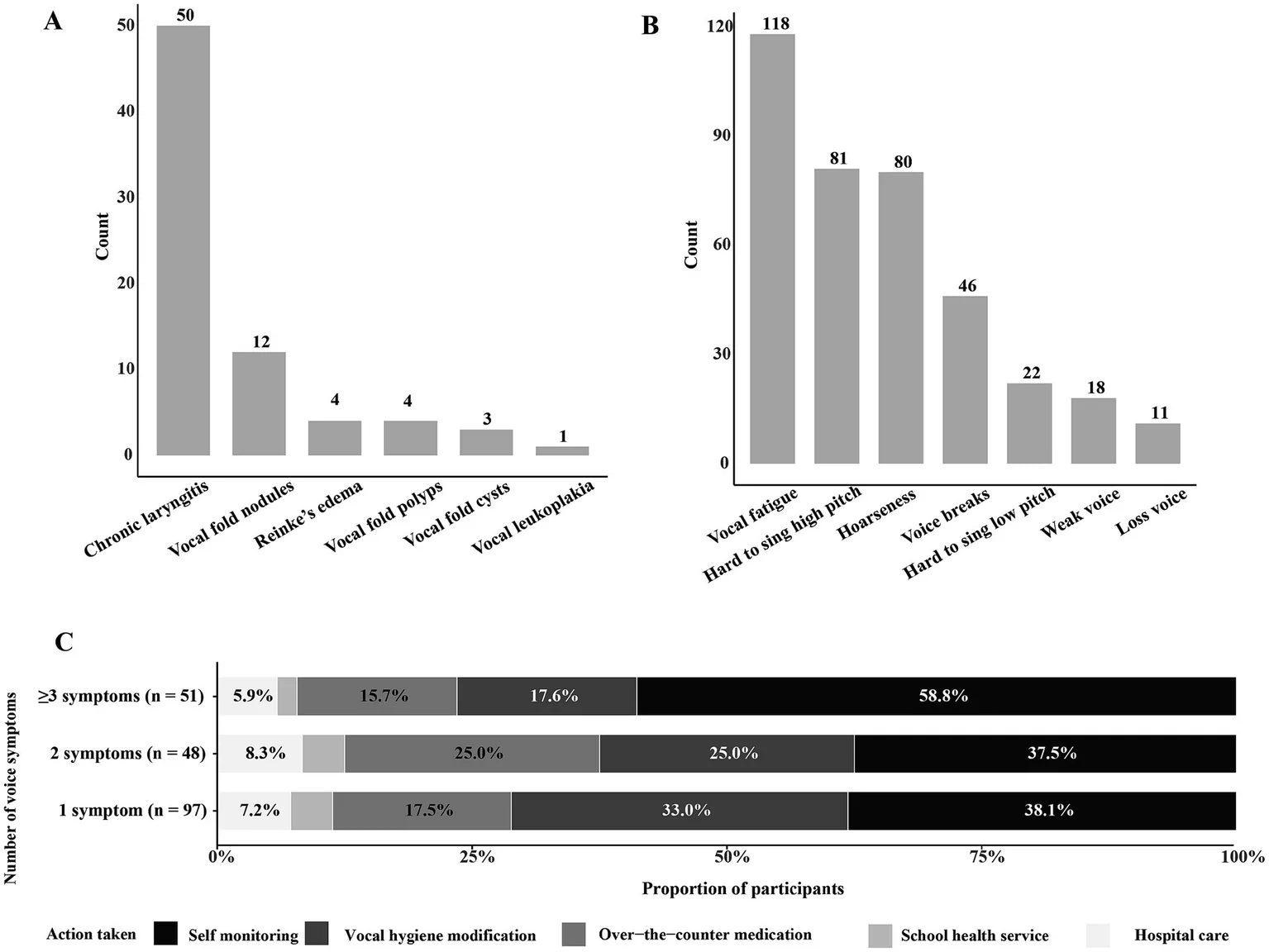
Self-reported voice symptoms and history of clinician-diagnosed voice disorders. **(A)** Previously diagnosed voice disorders. **(B)** Voice symptoms experienced during the preceding month. **(C)** Symptom-management behaviours among participants reporting one, two, or three or more symptoms.

A total of 119 participants (37.8%) reported no voice symptoms during the preceding month. Vocal fatigue was the most frequently reported symptom (*n* = 118; Figure 1B). Among the 196 participants reporting at least one symptom, 97 (49.5%) reported one symptom, 48 (24.5%) reported two symptoms, and 51 (26.0%) reported three or more symptoms. Fourteen symptomatic participants (7.1%) sought care at a municipal or higher-level hospital, and seven (3.6%) sought care through a school medical institution. The distribution of symptom-management behaviours did not differ across the three symptom-count groups (Fisher’s exact test, *p* = 0.360; Figure 1C).

The median self-rated voice-quality score was 7 (IQR, 6–8). Recent symptom count was negatively correlated with self-rated voice quality (Spearman’s *ρ* = −0.389, *p* < 0.001), indicating that participants reporting more symptoms tended to rate their voice quality less favourably.

### Vocal hygiene knowledge

The median vocal hygiene knowledge score was 8 (IQR, 7–9; range, 0–10). The proportion of participants who answered each item correctly ranged from 26.0 to 96.2%. The highest correct-response rate was observed for “Drinking plenty of water.” In contrast, “Frequent whispering” had both the lowest correct-response rate (26.0%) and the highest proportion of “Do not know” responses (40.0%), indicating a particularly prominent knowledge gap regarding the effects of whispering on vocal health (Figure 2).

**Figure 2:**
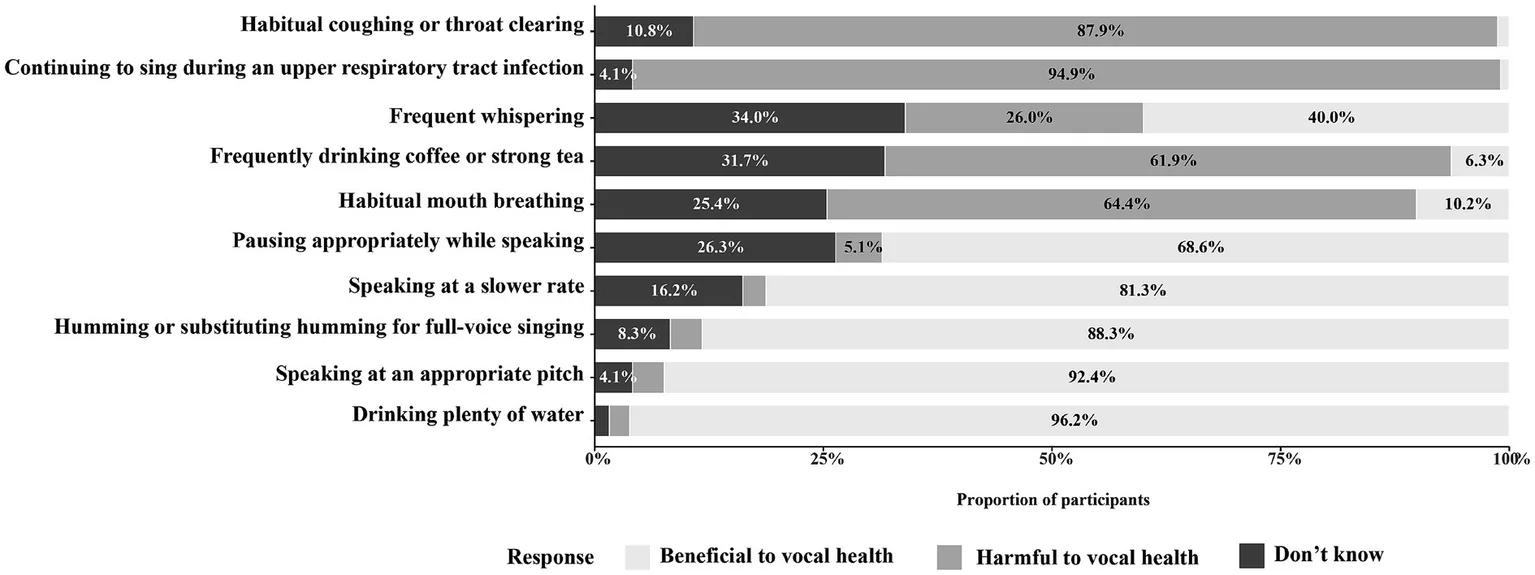
Distribution of responses to individual vocal hygiene knowledge items.

The multivariable linear regression model included age, gender, singing style, clinical exposure related to voice disorders, and recent symptom count. After adjustment for these covariates, female gender was associated with a higher vocal hygiene knowledge score (*β* = 0.792, 95% CI, 0.320–1.264; *p* = 0.001; Table 2).

**Table 2:** Factors associated with vocal hygiene knowledge in the multivariable linear regression model.

	*β*	95% CI, lower	95% CI, upper	Robust SE	*p* value
Gender					
Male	Reference				
Female	0.792	0.320	1.264	0.24	0.001
Age	−0.124	−0.278	0.03	0.078	0.113
Singing style					
Folk	Reference				
Classical	0.151	−0.354	0.656	0.257	0.557
Popular	0.580	−0.143	1.304	0.368	0.115
Voice disorders and treatment history					
No specific diagnosis reported	Reference				
Diagnosed, not treated	0.624	−0.071	1.319	0.353	0.078
Diagnosed and treated	0.056	−0.501	0.614	0.283	0.842
Number of voice symptoms	−0.105	−0.258	0.049	0.078	0.181

### Attitudes toward vocal hygiene

The median vocal hygiene attitude score was 7 (IQR, 5–8; range, −10 to 10). Most participants agreed or strongly agreed that vocal hygiene is extremely important for singers (*n* = 283, 89.8%), that appropriate vocal hygiene practices can prevent voice disorders (*n* = 269, 85.4%), and that vocal hygiene remains important in the absence of a voice disorder (*n* = 279, 88.6%). Similarly, 283 participants (89.8%) agreed or strongly agreed that early treatment is necessary when a voice disorder occurs. Responses regarding the ease of implementing vocal hygiene practices were more evenly distributed: 95 participants (30.2%) agreed or strongly agreed, 118 (37.5%) were neutral, and 102 (32.4%) disagreed or strongly disagreed. Knowledge and attitude scores showed a weak positive correlation (Spearman’s *ρ* = 0.143, *p* = 0.011; Figure 3).

**Figure 3:**
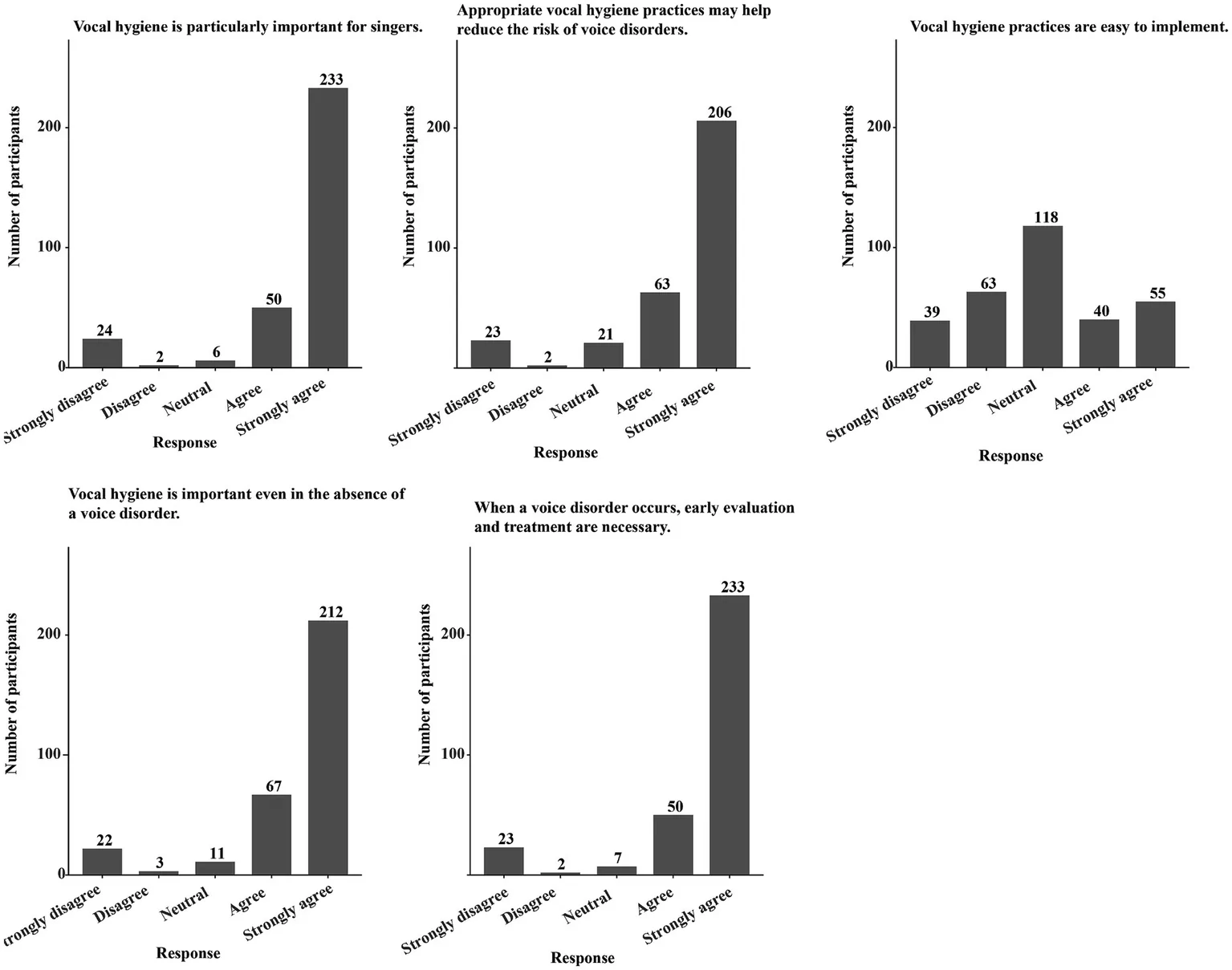
Distribution of responses to the five vocal hygiene attitude items.

### Voice-related practices

The most frequently reported potentially harmful voice-related practice was frequent throat clearing (*n* = 139, 44.1%). Warming up before singing was reported by 130 participants (41.3%), and cooling down after singing by 88 (27.9%).

Separate multivariable logistic regression models were fitted for the nine predefined voice-related practices among 315 participants. All models included the continuous knowledge score and clinical exposure and were adjusted for age, gender, singing style, and recent symptom count (Figure 4 and Supplementary Table 1). Each one-point increase in knowledge score was associated with higher odds of warming up before singing (aOR = 1.28, 95% CI, 1.11–1.47; *p* < 0.001; FDR-adjusted *p* = 0.006). A nominal association was also observed with cooling down after singing (aOR = 1.18, 95% CI, 1.02–1.38; *p* = 0.030), but it did not remain significant after correction for multiple comparisons (FDR-adjusted *p* = 0.133). Knowledge score was not associated with any of the seven potentially harmful practices. Compared with participants reporting no specific diagnosis, those who had been diagnosed and treated had higher odds of frequent throat clearing (aOR = 3.36, 95% CI, 1.71–6.58; *p* < 0.001; FDR-adjusted *p* = 0.004). A nominal association with speaking loudly (aOR = 2.07, 95% CI, 1.11–3.88; *p* = 0.023) did not remain significant after correction (FDR-adjusted *p* = 0.103). The “diagnosed, not treated” category was not associated with any of the nine practices. Across the seven potentially harmful practices, each additional voice symptom was associated with higher odds of reporting the practice (aOR range, 1.25–1.97; all FDR-adjusted *p* ≤ 0.016). The strongest association was observed for continuing to sing during an upper respiratory tract infection (aOR per additional symptom = 1.97, 95% CI, 1.55–2.50; *p* < 0.001). Female participants had higher odds of speaking loudly (aOR = 2.30, 95% CI, 1.31–4.04; *p* = 0.004; FDR-adjusted *p* = 0.017) and frequent shouting (aOR = 3.73, 95% CI, 1.72–8.08; *p* = 0.001; FDR-adjusted *p* = 0.007) than male participants. No associations involving age or singing style remained significant after FDR correction.

**Figure 4:**
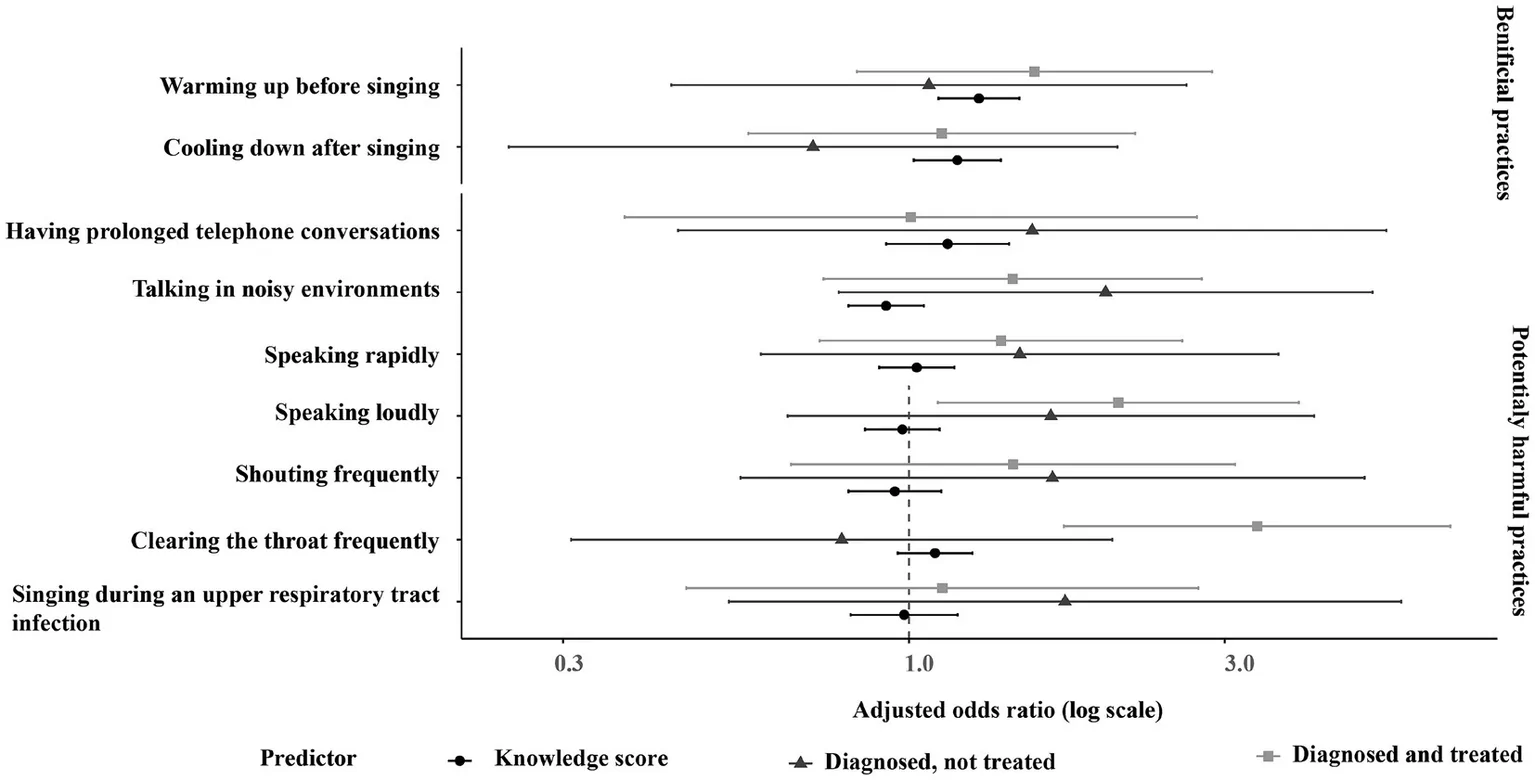
Adjusted associations of vocal hygiene knowledge and clinical exposure with individual voice-related practices.

### Educational needs and self-rated familiarity

Regarding self-rated familiarity with vocal hygiene, 120 participants (38.1%) selected “Acquainted,” whereas 51 (16.2%) selected either “Aware but unfamiliar” or “Never heard of it.” Self-rated familiarity showed a weak positive association with the objective knowledge score (Kendall’s τ-b = 0.189, *p* = 0.001).

Among the 273 participants interested in receiving further vocal hygiene information, the most frequently preferred channels were singing teachers (*n* = 229, 83.9%) and the school curriculum (*n* = 193, 70.7%). The most commonly requested topics were prevention of voice disorders (*n* = 243, 89.0%) and mechanisms of voice production (*n* = 202, 74.0%; Figures 5B,C). Because multiple responses were allowed, percentages do not sum to 100%.

**Figure 5:**
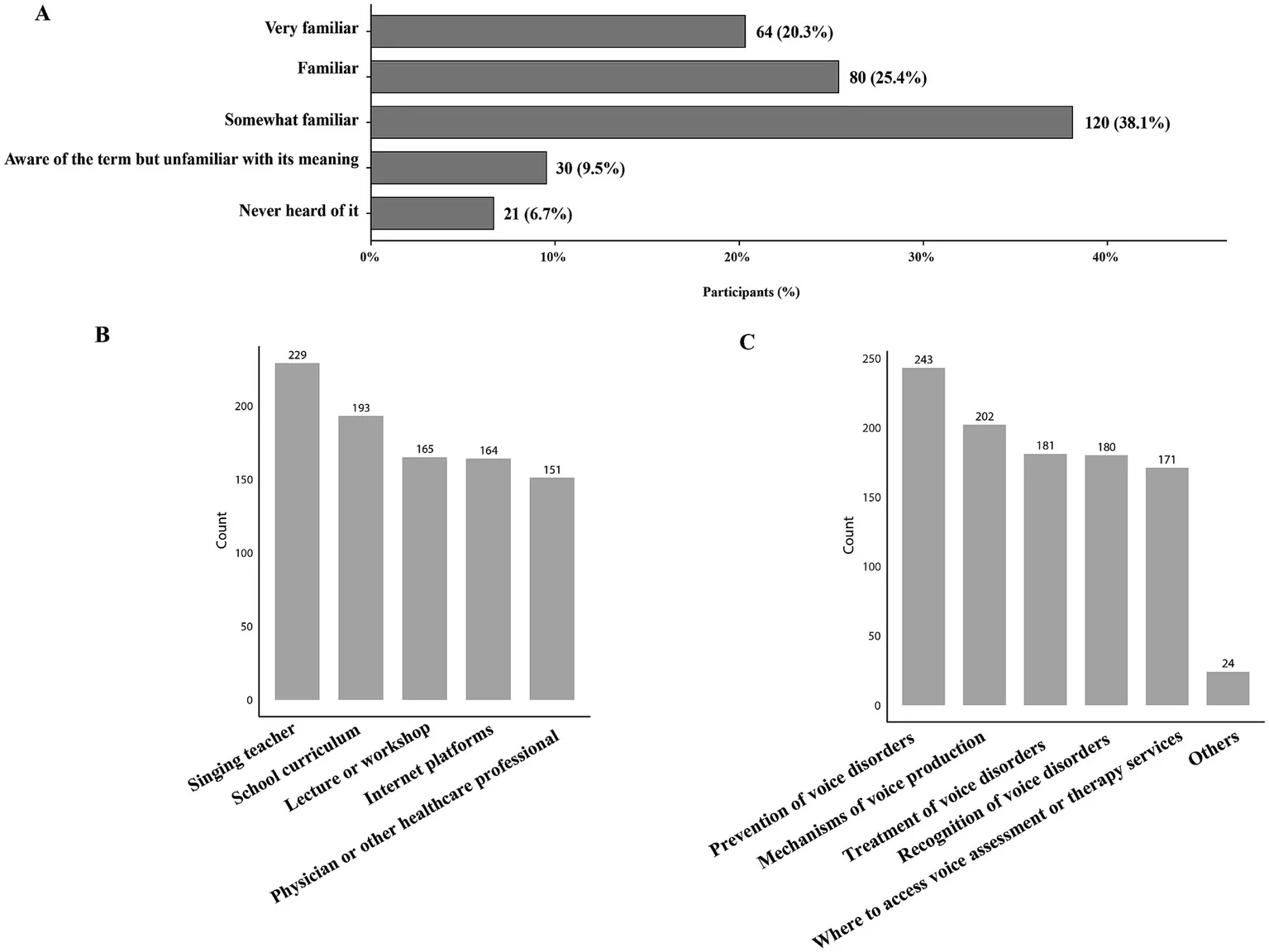
Vocal hygiene familiarity and educational needs among conservatory students. **(A)** Self-rated familiarity with vocal hygiene (*N* = 315). **(B)** Preferred channels for obtaining vocal hygiene information (*n* = 273). **(C)** Types of vocal hygiene information desired (*n* = 273).

## Discussion

This cross-sectional survey identified a clear mismatch between generally favourable vocal hygiene knowledge and attitudes and the implementation of appropriate voice-related practices among Chinese conservatory students. Although the median knowledge score was 8 out of 10 and the median attitude score was 7 on a scale ranging from −10 to 10, fewer than half of the participants reported warming up before singing, fewer than one-third reported cooling down afterwards, and frequent throat clearing remained common. Higher knowledge was associated with warming up before singing but not with any of the seven potentially harmful practices. The central implication is therefore not simply that conservatory students require more information, but that specific misconceptions must be corrected and existing knowledge must be translated into routines that can be sustained during singing, rehearsal and everyday voice use.

The generally favourable knowledge observed in this study differs to some extent from findings in other populations of voice users. Young classical singers enrolled in elite training programmes in the United States obtained widely varying knowledge scores, with no clear improvement across successive levels of training, although most expressed a strong interest in learning more about vocal anatomy, pathology and care (7). Brazilian student teachers similarly demonstrated insufficient knowledge of vocal health and hygiene (12), while a survey of occupational voice users in India identified limited awareness and unfavourable attitudes in several occupational groups (13). More recently, a Chinese survey of occupational voice users reported moderate knowledge and practices and relatively neutral attitudes toward vocal fatigue (21). Compared with these populations, the conservatory students in the present study appeared to have a stronger general knowledge base and more favourable attitudes, but this relative strength coexisted with substantial item-specific and behavioural gaps. Nevertheless, the common finding is that participation in a voice-intensive profession or training programme does not itself ensure comprehensive vocal-health knowledge.

The item-level results are particularly informative in this respect. “Frequent whispering” had both the lowest correct-response rate and the highest proportion of “Do not know” responses, despite the relatively high overall knowledge score. Thus, a total score alone may obscure misconceptions that are clinically or behaviourally relevant. The weak association between objective knowledge scores and self-rated familiarity further suggests that students may not accurately identify the limits of their own understanding. Vocal-health education should therefore not be planned solely on the basis of students’ perceived familiarity or overall scores. Brief diagnostic assessments at the beginning of a course could instead be used to identify misconceptions concerning whispering, throat clearing, singing during upper respiratory tract infection, vocal load and recovery, and other behaviours that may otherwise be overlooked.

The weak positive relationship between knowledge and attitudes, together with the limited association between knowledge and individual practices, also illustrates that the three KAP domains should not be assumed to form a simple linear sequence. A recent study of occupational voice users in China found positive associations among aggregate knowledge, attitude and practice score (21). In the present study, however, analysing individual behaviours separately revealed that knowledge was associated with warm-up but not with the seven potentially harmful practices. This difference may partly reflect variations in vocal demands across populations and in the measurement of practices. Studies using a composite practice score may identify an overall association with knowledge even when knowledge is related to only some behaviours. By analysing each practice separately, our study showed that higher knowledge was associated with vocal warm-up but not with the potentially harmful practices, indicating that favourable knowledge does not necessarily translate into consistently appropriate daily voice use.

One possible explanation is that warm-up is closely integrated with formal singing instruction and may therefore be repeatedly cued by lessons, rehearsals and performances. By contrast, throat clearing, loud speech, rapid speech and prolonged telephone use often occur outside formal singing contexts, where students receive fewer prompts and may have less control over environmental demands. This distinction may explain why knowledge was translated into warm-up behaviour but not into reductions in potentially harmful habits. Small studies among singing students have reported favourable acoustic changes following warm-up and cool-down procedures (22), but the optimal content, duration and long-term protective effects of these routines remain uncertain. Accordingly, conservatory education should frame warm-up and cool-down as structured skills that require demonstration and supervised practice, rather than presenting them only as recommended concepts.

The association between a greater number of recent voice symptoms and all seven potentially harmful practices warrants particular attention. This pattern is compatible with the possibility that repeated harmful voice use contributes to symptom development. However, the cross-sectional design also permits reverse or bidirectional explanations: students experiencing symptoms may become more aware of their behaviour, may report it more readily, or may adopt compensatory voice-use patterns that increase laryngeal effort. Similarly, the association between previous diagnosis and treatment and frequent throat clearing should not be interpreted as an adverse effect of treatment. Throat clearing may have preceded diagnosis, reflected an underlying laryngeal condition, or persisted after treatment. These findings support teaching students to recognise symptom–behaviour cycles without implying blame or causality. For example, education could encourage students to respond to recurrent throat discomfort by reducing vocal load, using appropriate alternatives to forceful throat clearing and seeking assessment when symptoms persist, rather than continuing the behaviour that may accompany or aggravate discomfort.

The reported symptom burden should also be interpreted cautiously. The 62.2% of participants reporting at least one recent symptom cannot be equated with the prevalence of clinically confirmed voice disorders or directly compared with pooled dysphonia prevalence estimates among singers (5). Nevertheless, the negative correlation between symptom count and self-rated voice quality indicates that the symptoms were meaningfully related to perceived vocal function. The combination of frequent symptoms, potentially harmful practices and limited use of professional care therefore identifies an important area for prevention and early intervention.

Only 21 of the 196 symptomatic participants reported seeking care from a school medical institution or hospital. This tendency to avoid professional healthcare resembles concerns reported in other performing populations. Young classical singers have reported anxiety about laryngeal disorders and clinical visits (7), while performers with previous voice injuries have described uneven access to specialist resources, variable organisational support and concerns about discussing voice injuries (23). A qualitative study of Carnatic singers further identified reliance on home remedies or modification of singing technique, uncertainty about voice-therapy outcomes, financial and logistical barriers, and strong influence from singing teacher (24). Although these findings arise from different cultural and healthcare settings, they suggest that delayed care may reflect more than insufficient knowledge. Students may lack a clear referral pathway, may fear that diagnosis will interrupt training, or may initially interpret symptoms as a technical problem to be managed only with their singing teacher.

These findings indicate three complementary areas for future vocal-health education. First, foundational content should target misconceptions and include vocal anatomy, vocal-load management, hydration, throat-clearing alternatives, voice use during respiratory infection, early warning symptoms and indications for professional assessment. Second, knowledge should be converted into observable skills through demonstrations, guided warm-up and cool-down routines, rehearsal-based scenarios, self-monitoring and individual action plans. For example, students could formulate situation-specific plans such as reducing vocal load and contacting a designated service if hoarseness persists beyond a defined period. Third, education should include a clear care pathway distinguishing the complementary roles of singing teachers, otolaryngologists and speech-language therapists. Singing teachers may help reinforce healthy routines and recognise deviations from a student’s usual voice, but they should not be expected to diagnose laryngeal pathology.

The preferred educational channels reported by participants provide a feasible basis for this approach. Most interested students selected singing teachers and the school curriculum, suggesting that vocal-health content could be embedded within existing pedagogical relationships rather than delivered only through isolated extracurricular lectures. Evidence from performing-arts students suggests that programmes combining theoretical instruction with direct voice exercises may improve knowledge and some voice outcomes, although available studies remain small (25). Systematic reviews likewise indicate that vocal hygiene education produces mixed results when delivered alone and may be more useful when combined with direct voice training or therapy (10, 11). A blended model may therefore be appropriate: a core curriculum delivered at entry to conservatory training, brief reinforcement within individual lessons and rehearsals, practical workshops led jointly by singing teachers and voice-care professionals, and periodic digital reminders or self-monitoring tools. Mobile vocal-health resources have shown high usability and acceptability among teachers (26), but their effectiveness among conservatory students and their ability to change behaviour require direct evaluation.

Educational content should also be sensitive to vocal genre without assuming that one singing tradition is inherently more harmful than another. A shared core curriculum could address principles common to folk, classical and popular singing, whereas practice scenarios could be adapted to differences in rehearsal load, amplification, register use, performance environment and stylistic voice production. The absence of an adjusted association between singing style and the measured practices suggests that separate programmes for each genre are not yet justified by the present data. Similarly, although female participants had higher adjusted knowledge scores, this finding alone does not support gender-specific education. Future programmes would be better tailored according to baseline misconceptions, symptom patterns and actual vocal demands than according to demographic characteristics alone.

Future studies should evaluate not only whether an educational programme increases knowledge, but whether it changes behaviour and improves access to appropriate care. A pragmatic trial could compare lecture-only education with a blended programme incorporating skills practice, teacher reinforcement and digital follow-up. Relevant outcomes would include item-level knowledge retention, objectively or prospectively recorded voice-related practices, symptom trajectories, use of professional services, validated patient-reported voice measures and, where feasible, acoustic or clinical voice assessments. Longer follow-up would be necessary to determine whether early behavioural changes persist during examinations, intensive rehearsal periods and performances. Such studies would move beyond documenting educational demand and establish which components are effective, for whom and under what training conditions.

Taken together, the findings suggest that conservatory students do not primarily face a uniform deficiency in vocal hygiene knowledge. Rather, they show a combination of specific misconceptions, incomplete transfer of knowledge into daily behaviour and limited engagement with professional voice care. Addressing these gaps will probably require repeated, practice-oriented and context-sensitive education supported by teachers and accessible clinical referral pathways, rather than a single increase in the amount of general vocal-health information.

## Limitations

Several limitations should be considered. First, participants were recruited through convenience sampling and completed a voluntary online questionnaire. The sample was geographically diverse but was not selected using a nationally representative sampling framework, and students with greater interest in vocal health may have been more likely to participate. The findings therefore may not be generalizable to all conservatory students in mainland China. Second, voice symptoms, previous diagnoses, treatments and voice-related practices were self-reported. Differences in symptom awareness, recall and interpretation may have introduced reporting bias. Previous diagnoses were not verified through medical records or laryngeal examinations, and many participants had never sought medical evaluation. Consequently, the study cannot provide an estimate of the prevalence of clinically confirmed voice disorders. Third, although the questionnaire was developed with reference to previous studies and reviewed by relevant experts, its psychometric properties, including construct validity and test–retest reliability, were not formally evaluated. Moreover, no speech-language pathologist (SLP) or voice therapist was included in the expert panel because no professional with specialised voice-therapy expertise was available within the collaborating institutions. Given the central role of SLPs in vocal hygiene assessment and intervention, this omission may have limited the questionnaire’s content validity and clinical framing. Future studies should include SLPs or voice therapists in questionnaire development and conduct formal psychometric validation. Fourth, the 20 participants involved in pilot testing were retained in the final analytic sample, although pilot participants would ideally be excluded from the main analysis. This pragmatic decision was made to preserve the attainable sample size and study feasibility, given that pilot testing resulted only in minor wording clarification and did not alter the constructs assessed, response categories, scoring, or questionnaire structure. Nevertheless, including responses collected using a preliminary version of the questionnaire may have introduced some measurement heterogeneity and should be considered when interpreting the findings. Finally, the cross-sectional design precludes conclusions about temporal or causal relationships between knowledge, attitudes, symptoms and voice-related practices. The regression analyses identified associations rather than determinants or effects of vocal hygiene education. Some clinical and vocal-range categories also contained few participants, which may have reduced statistical precision. Prospective studies incorporating validated questionnaires, objective voice assessments and longitudinal follow-up are needed to determine how knowledge and practices relate to subsequent vocal outcomes.

## Conclusion

Chinese conservatory students demonstrated generally favourable vocal hygiene knowledge and attitudes, but specific knowledge gaps and inconsistencies in voice-related practices remained. Frequent whispering was particularly poorly understood, and higher knowledge was associated with warming up before singing but not with potentially harmful practices overall. A substantial proportion of participants reported recent voice symptoms, while relatively few sought professional healthcare. These findings support refining conservatory-based vocal health education to emphasise overlooked concepts, practical behaviour implementation, symptom recognition and appropriate healthcare-seeking pathways.

## Data Availability

The raw data supporting the conclusions of this article will be made available by the authors upon reasonable request, subject to ethical and privacy considerations.
