# Supplementary material for: Knowledge, attitudes, and practice patterns related to vocal hygiene among conservatory students in China
Source: Front Public Health. 2026 Oct 7;14:1942292. doi: 10.3389/fpubh.2026.1942292 (PMC13683749; doi:10.3389/fpubh.2026.1942292)
Supplement: Supplementary file 1 [file Table_1.DOCX]

**Supplementary Appendix 1.** English translation of the questionnaire administered to participants

**Section I: Participant characteristics**

1. Sex:

☐ Male

☐ Female

2. Age: _____ years

3. Primary singing style:

☐ Chinese folk singing

☐ Western classical singing

☐ Popular singing

4. Your vocal range:

☐ High
☐ Medium
☐ Low

**Section II: History of voice disorders and voice symptoms**

5. Have you ever been diagnosed with any of the following voice disorders? Select all that apply. *If either of the first two options applies, select only that option. Otherwise, select all diagnosed conditions that apply.*

☐ I have never sought medical evaluation for a voice problem (**Skip Question 6**)

☐ I sought medical evaluation, but no voice disorder was diagnosed (**Skip Question 6**)

☐ Vocal fold nodules

☐ Vocal fold polyps

☐ Chronic laryngitis

☐ Vocal fold cysts

☐ Reinke’s edema

☐ Vocal leukoplakia

☐ Sulcus vocalis

☐ Vocal fold paralysis

☐ Other: _____________

6. Have you ever received any treatment for a diagnosed voice disorder, including medication, surgery, or voice therapy?

☐ Yes

☐ No

7. During the past month, have you experienced any of the following voice symptoms? Select all that apply.

☐ No symptom (**Skip Question 8**)

☐ Hoarseness

☐ Weak voice

☐ Vocal fatigue

☐ Voice breaks

☐ Loss voice

☐ Hard to sing high pitch

☐ Hard to sing low pitch

☐ Others: _____________

8. If you experienced any of the above voice symptoms, what was the main action you took? Please select one option.

☐ Monitored the symptoms and waited for them to improve without treatment

☐ Modified my vocal hygiene practices

☐ Took over-the-counter medication

☐ Sought care from the school health service

☐ Consulting a municipal or higher-level hospital

9. How would you rate your overall voice quality during the past month? (1 = Very Poor, 10 = Excellent)

**Section III: Vocal hygiene knowledge**

10. Based on your current knowledge, please indicate whether you consider each of the following voice-related behaviors or habits to be beneficial or harmful to vocal health. Select “Don’t know” if you are unsure.

| Items | Options | | |
| --- | --- | --- | --- |
|  | Beneficial to vocal health | Harmful to vocal health | Don’t know |
| Habitual coughing or throat clearing |  | 🗸 |  |
| Continuing to sing during an upper respiratory tract infection |  | 🗸 |  |
| Speaking at an appropriate pitch | 🗸 |  |  |
| Frequently drinking coffee or strong tea |  | 🗸 |  |
| Speaking at a slower rate， | 🗸 |  |  |
| Pausing appropriately while speaking | 🗸 |  |  |
| Habitual mouth breathing |  | 🗸 |  |
| Humming or substituting humming for full-voice singing | 🗸 |  |  |
| Frequent whispering |  | 🗸 |  |
| Drinking plenty of water | 🗸 |  |  |

**Section IV: Attitudes toward vocal hygiene**

11. Please select your views on vocal hygiene:

| Items | Options | | | | |
| --- | --- | --- | --- | --- | --- |
|  | Strongly disagree | Disagree | Neutral | Agree | Strongly agree |
| When a voice disorder occurs, early evaluation and treatment are necessary. |  |  |  |  |  |
| Vocal hygiene is important even in the absence of a voice disorder. |  |  |  |  |  |
| Vocal hygiene practices are easy to implement. |  |  |  |  |  |
| Appropriate vocal hygiene practices may help reduce the risk of voice disorders. |  |  |  |  |  |
| Vocal hygiene is particularly important for singers. |  |  |  |  |  |

**Section V: Voice-related practices**

12. During the past month, did you engage in any of the following voice-related practices? Select all that apply.

☐ Having prolonged telephone conversations

☐ Talking in noisy environments

☐ Speaking rapidly

☐ Speaking loudly

☐ Shouting frequently

☐ Clearing the throat frequently

☐ Singing during an upper respiratory tract infection

☐ Warming up before singing

☐ Cooling down after singing

☐ Other: _____________

☐ None of the above

**Section VI: Vocal hygiene educational needs**

13. How familiar are you with vocal hygiene?

*Vocal hygiene refers to behaviours and environmental measures intended to maintain vocal health and reduce avoidable vocal strain, including appropriate voice use, hydration, management of vocal load, and avoidance of potentially phonotraumatic behaviours.*

☐ Never heard of it

☐ Aware of the term but unfamiliar with its meaning

☐ Somewhat familiar

☐ Familiar

☐ Very familiar

14. Are you interested in receiving further information about vocal hygiene?

☐ Yes

☐ No (skip 15,16)

15. What types of vocal hygiene information would you like to receive? Select all that apply.

☐ Prevention of voice disorders

☐ Mechanisms of voice production

☐ Recognition of voice disorders

☐ Treatment of voice disorders

☐ Where to access voice assessment or therapy services

☐ Other: _____________

16. Through which channels would you prefer to receive vocal hygiene information? Select all that apply.

☐ Singing teacher

☐ School curriculum

☐ Physician or other healthcare professional

☐ Lecture or workshop

☐ Internet platforms, such as WeChat, Baidu, Xiaohongshu, or Douyin

☐ Other: _____________

**Supplementary Table 1.**

Multivariable logistic regression analyses of factors associated with the nine voice-related practices

| Outcome | N | Factor | Estimate | Std.error | Adjusted_OR | CI_lower | CI_upper | P value | FDR-adjusted P | |
| --- | --- | --- | --- | --- | --- | --- | --- | --- | --- | --- |
| Long telephone conversations | 315 | Knowledge_score | 0.134416 | 0.10915 | 1.143869 | 0.923561 | 1.416729 | 0.218145 | 0.42172 |  |
| Long telephone conversations | 315 | Diagnosed, not treated | 0.428488 | 0.628657 | 1.534935 | 0.447678 | 5.262765 | 0.495497 | 0.689516 |  |
| Long telephone conversations | 315 | Diagnosed and treated | 0.006022 | 0.508032 | 1.00604 | 0.37168 | 2.723083 | 0.990543 | 0.990543 |  |
| Long telephone conversations | 315 | Age | 0.364503 | 0.158061 | 1.439799 | 1.056228 | 1.962662 | 0.021105 | 0.094973 |  |
| Long telephone conversations | 315 | Female | 1.099333 | 0.460336 | 3.002163 | 1.217835 | 7.400823 | 0.016935 | 0.050806 |  |
| Long telephone conversations | 315 | Western classical singing | 0.427586 | 0.422761 | 1.533552 | 0.669633 | 3.512045 | 0.311819 | 0.940285 |  |
| Long telephone conversations | 315 | Popular singing | -2.06811 | 1.12574 | 0.126425 | 0.013918 | 1.148372 | 0.066194 | 0.254826 |  |
| Long telephone conversations | 315 | Number of voice symptoms | 0.325881 | 0.129246 | 1.38525 | 1.075255 | 1.784617 | 0.011689 | 0.01559 |  |
| Talking in noisy settings | 315 | Knowledge_score | -0.07963 | 0.066949 | 0.92346 | 0.809898 | 1.052946 | 0.234289 | 0.42172 |  |
| Talking in noisy settings | 315 | Diagnosed, not treated | 0.684024 | 0.473698 | 1.981837 | 0.783156 | 5.015193 | 0.148736 | 0.689516 |  |
| Talking in noisy settings | 315 | Diagnosed and treated | 0.360452 | 0.335654 | 1.433977 | 0.742725 | 2.768575 | 0.282876 | 0.53762 |  |
| Talking in noisy settings | 315 | Age | 0.174335 | 0.100772 | 1.190454 | 0.977089 | 1.450411 | 0.08363 | 0.188168 |  |
| Talking in noisy settings | 315 | Female | -0.00586 | 0.287944 | 0.994159 | 0.565397 | 1.748069 | 0.983769 | 0.990627 |  |
| Talking in noisy settings | 315 | Western classical singing | -0.42005 | 0.296453 | 0.657013 | 0.367475 | 1.174681 | 0.156505 | 0.940285 |  |
| Talking in noisy settings | 315 | Popular singing | -0.63746 | 0.464427 | 0.528633 | 0.212728 | 1.313658 | 0.169884 | 0.254826 |  |
| Talking in noisy settings | 315 | Number of voice symptoms | 0.402607 | 0.095615 | 1.495719 | 1.240113 | 1.804009 | 2.55E-05 | 8.36E-05 |  |
| Talking rapidly | 315 | Knowledge_score | 0.026994 | 0.066866 | 1.027362 | 0.901169 | 1.171226 | 0.686425 | 0.81625 |  |
| Talking rapidly | 315 | Diagnosed, not treated | 0.385396 | 0.459189 | 1.470197 | 0.597731 | 3.616136 | 0.401302 | 0.689516 |  |
| Talking rapidly | 315 | Diagnosed and treated | 0.319853 | 0.321911 | 1.376925 | 0.732645 | 2.587776 | 0.320415 | 0.53762 |  |
| Talking rapidly | 315 | Age | -0.0677 | 0.099712 | 0.934539 | 0.768637 | 1.13625 | 0.497155 | 0.6392 |  |
| Talking rapidly | 315 | Female | 0.400298 | 0.275028 | 1.492269 | 0.870439 | 2.558326 | 0.145536 | 0.218304 |  |
| Talking rapidly | 315 | Western classical singing | 0.080629 | 0.279162 | 1.083969 | 0.627176 | 1.873459 | 0.772714 | 0.940285 |  |
| Talking rapidly | 315 | Popular singing | -0.77689 | 0.472618 | 0.459833 | 0.182096 | 1.161185 | 0.100217 | 0.254826 |  |
| Talking rapidly | 315 | Number of voice symptoms | 0.224904 | 0.089658 | 1.252203 | 1.050403 | 1.492771 | 0.012126 | 0.01559 |  |
| Speaking loudly | 315 | Knowledge_score | -0.02324 | 0.066195 | 0.97703 | 0.858147 | 1.112384 | 0.725556 | 0.81625 |  |
| Speaking loudly | 315 | Diagnosed, not treated | 0.493548 | 0.467273 | 1.638118 | 0.655532 | 4.093512 | 0.290863 | 0.689516 |  |
| Speaking loudly | 315 | Diagnosed and treated | 0.728359 | 0.320088 | 2.071678 | 1.106261 | 3.879599 | 0.022876 | 0.102943 |  |
| Speaking loudly | 315 | Age | 0.068911 | 0.098045 | 1.071341 | 0.884037 | 1.29833 | 0.482144 | 0.6392 |  |
| Speaking loudly | 315 | Female | 0.832927 | 0.287101 | 2.300041 | 1.310239 | 4.037577 | 0.003718 | 0.01673 |  |
| Speaking loudly | 315 | Western classical singing | 0.124592 | 0.285543 | 1.132686 | 0.647218 | 1.982298 | 0.662594 | 0.940285 |  |
| Speaking loudly | 315 | Popular singing | -0.28038 | 0.444026 | 0.755495 | 0.316423 | 1.803826 | 0.527744 | 0.593712 |  |
| Speaking loudly | 315 | Number of voice symptoms | 0.293822 | 0.091869 | 1.341545 | 1.120482 | 1.606222 | 0.001383 | 0.002489 |  |
| Frequent shouting | 315 | Knowledge_score | -0.04954 | 0.082399 | 0.95167 | 0.809743 | 1.118473 | 0.547715 | 0.81625 |  |
| Frequent shouting | 315 | Diagnosed, not treated | 0.499287 | 0.553833 | 1.647547 | 0.556423 | 4.87832 | 0.367316 | 0.689516 |  |
| Frequent shouting | 315 | Diagnosed and treated | 0.362023 | 0.394192 | 1.436233 | 0.663256 | 3.110056 | 0.358413 | 0.53762 |  |
| Frequent shouting | 315 | Age | -0.04244 | 0.134929 | 0.958447 | 0.735723 | 1.248595 | 0.753107 | 0.847245 |  |
| Frequent shouting | 315 | Female | 1.317212 | 0.394108 | 3.733 | 1.724197 | 8.082192 | 0.000831 | 0.007479 |  |
| Frequent shouting | 315 | Western classical singing | 0.317495 | 0.351941 | 1.373682 | 0.68914 | 2.738196 | 0.366991 | 0.940285 |  |
| Frequent shouting | 315 | Popular singing | -1.23417 | 0.720189 | 0.291076 | 0.070953 | 1.194108 | 0.086588 | 0.254826 |  |
| Frequent shouting | 315 | Number of voice symptoms | 0.434794 | 0.10711 | 1.544645 | 1.252146 | 1.905471 | 4.92E-05 | 0.000111 |  |
| Frequent throat clearing | 315 | Knowledge_score | 0.090421 | 0.066319 | 1.094635 | 0.961208 | 1.246583 | 0.172751 | 0.42172 |  |
| Frequent throat clearing | 315 | Diagnosed, not treated | -0.23417 | 0.480452 | 0.791225 | 0.308554 | 2.028939 | 0.625974 | 0.704221 |  |
| Frequent throat clearing | 315 | Diagnosed and treated | 1.211123 | 0.342963 | 3.357252 | 1.714145 | 6.575373 | 0.000413 | 0.003721 |  |
| Frequent throat clearing | 315 | Age | 0.225472 | 0.097442 | 1.252914 | 1.035088 | 1.516579 | 0.020672 | 0.094973 |  |
| Frequent throat clearing | 315 | Female | -0.54432 | 0.271443 | 0.580238 | 0.340839 | 0.987788 | 0.044935 | 0.101103 |  |
| Frequent throat clearing | 315 | Western classical singing | -0.02338 | 0.286121 | 0.976891 | 0.557564 | 1.711581 | 0.934874 | 0.940285 |  |
| Frequent throat clearing | 315 | Popular singing | -0.65978 | 0.442429 | 0.516964 | 0.217199 | 1.230449 | 0.13589 | 0.254826 |  |
| Frequent throat clearing | 315 | Number of voice symptoms | 0.412734 | 0.098499 | 1.510943 | 1.245672 | 1.832705 | 2.79E-05 | 8.36E-05 |  |
| Singing during an upper respiratory infection | 315 | Knowledge_score | -0.01684 | 0.094954 | 0.983302 | 0.816319 | 1.184441 | 0.859241 | 0.859241 |  |
| Singing during an upper respiratory infection | 315 | Diagnosed, not treated | 0.542966 | 0.596694 | 1.721103 | 0.53443 | 5.542722 | 0.362845 | 0.689516 |  |
| Singing during an upper respiratory infection | 315 | Diagnosed and treated | 0.116039 | 0.454569 | 1.12304 | 0.460742 | 2.737361 | 0.798513 | 0.898327 |  |
| Singing during an upper respiratory infection | 315 | Age | 0.265499 | 0.146909 | 1.304082 | 0.977808 | 1.739226 | 0.070725 | 0.188168 |  |
| Singing during an upper respiratory infection | 315 | Female | 0.393053 | 0.408272 | 1.481497 | 0.665538 | 3.297835 | 0.335686 | 0.431596 |  |
| Singing during an upper respiratory infection | 315 | Western classical singing | 0.245638 | 0.41655 | 1.278437 | 0.565074 | 2.892368 | 0.555393 | 0.940285 |  |
| Singing during an upper respiratory infection | 315 | Popular singing | -1.13123 | 0.782871 | 0.322637 | 0.069554 | 1.496606 | 0.148465 | 0.254826 |  |
| Singing during an upper respiratory infection | 315 | Number of voice symptoms | 0.676446 | 0.122203 | 1.966875 | 1.547944 | 2.499185 | 3.10E-08 | 2.79E-07 |  |
| Warming up before singing | 315 | Knowledge_score | 0.243091 | 0.071632 | 1.275184 | 1.10815 | 1.467397 | 0.00069 | 0.006208 |  |
| Warming up before singing | 315 | Diagnosed, not treated | 0.069161 | 0.457091 | 1.071609 | 0.437474 | 2.624943 | 0.879733 | 0.879733 |  |
| Warming up before singing | 315 | Diagnosed and treated | 0.43647 | 0.315274 | 1.547236 | 0.834047 | 2.87027 | 0.166231 | 0.498692 |  |
| Warming up before singing | 315 | Age | -0.01685 | 0.090951 | 0.983288 | 0.822739 | 1.175166 | 0.852991 | 0.852991 |  |
| Warming up before singing | 315 | Female | -0.40229 | 0.260936 | 0.668784 | 0.401026 | 1.115318 | 0.123137 | 0.218304 |  |
| Warming up before singing | 315 | Western classical singing | -0.02038 | 0.272121 | 0.979821 | 0.574795 | 1.670247 | 0.940285 | 0.940285 |  |
| Warming up before singing | 315 | Popular singing | -0.29977 | 0.410171 | 0.740986 | 0.331638 | 1.655597 | 0.46487 | 0.593712 |  |
| Warming up before singing | 315 | Number of voice symptoms | -0.05604 | 0.089112 | 0.945505 | 0.793981 | 1.125945 | 0.529459 | 0.529459 |  |
| Cooling down after singing | 315 | Knowledge_score | 0.167875 | 0.077145 | 1.182789 | 1.016811 | 1.375861 | 0.029547 | 0.132964 |  |
| Cooling down after singing | 315 | Diagnosed, not treated | -0.33408 | 0.540209 | 0.715994 | 0.248356 | 2.064167 | 0.53629 | 0.689516 |  |
| Cooling down after singing | 315 | Diagnosed and treated | 0.113729 | 0.343301 | 1.120448 | 0.5717 | 2.195917 | 0.740433 | 0.898327 |  |
| Cooling down after singing | 315 | Age | -0.1074 | 0.10184 | 0.898165 | 0.735645 | 1.096589 | 0.291602 | 0.524883 |  |
| Cooling down after singing | 315 | Female | -0.00332 | 0.282603 | 0.996685 | 0.572799 | 1.73426 | 0.990627 | 0.990627 |  |
| Cooling down after singing | 315 | Western classical singing | 0.171618 | 0.295936 | 1.187224 | 0.664702 | 2.120501 | 0.561972 | 0.940285 |  |
| Cooling down after singing | 315 | Popular singing | -0.16701 | 0.457187 | 0.846193 | 0.345385 | 2.073173 | 0.714893 | 0.714893 |  |
| Cooling down after singing | 315 | Number of voice symptoms | -0.13357 | 0.102421 | 0.874969 | 0.71583 | 1.069487 | 0.192202 | 0.216227 |  |
